# Elucidating the Structure–Nonlinear Optical Property Relationship of Ethynyl Extended Benzanthrone Chromophores

**DOI:** 10.3390/molecules31091467

**Published:** 2026-04-28

**Authors:** Divya Jattu Gouda, B. Siddlingeshwar, H. M. Suresh Kumar, Shivaraj R. Maidur, S. R. Manohara, Armands Maleckis, Elena M. Kirilova

**Affiliations:** 1 Department of Physics, M.S. Ramaiah Institute of Technology (Autonomous Institute Affiliated to VTU), Bengaluru 560054, India; 2 Department of Physics, Siddaganga Institute of Technology (Autonomous Institute Affiliated to VTU), Tumakuru 572103, India; 3 Department of Physics, School of Computational & Physical Sciences, Kristu Jayanti (Deemed to be University), Bengaluru 560077, India; 4 Institute of Life Sciences and Technology, Daugavpils University, Vienibas 13, LV-5401 Daugavpils, Latvia

**Keywords:** benzanthrone dyes, ethynyl derivatives, fluorescence, nonlinear optics

## Abstract

Three ethynyl-extended benzanthrone derivatives with benzonitrile (Dye A), thiophene (Dye B), and methyl propiolate (Dye C) as substituents were synthesized and investigated to illustrate structure–property relationships governing their nonlinear optical (NLO) behavior. The third-order nonlinear absorption and refractive index of three dyes were studied using open- and closed-aperture z-scan measurements under 532 nm continuous-wave laser excitation. All dyes exhibited reverse saturable absorption dominated by two-photon absorption, with Dye A showing the highest nonlinear absorption coefficient (βeff = 2.3 × 10^−5^ cm/W) and two-photon response, attributed to its extended conjugation and smaller HOMO−LUMO gap (6.45 eV). Closed-aperture Z-scans revealed strong nonlinear refraction (n_2_), with the thiophene-substituted Dye B displaying the largest n_2_ (14.8 × 10^−9^ cm^2^/W) and third-order susceptibility (χ^3^ = 3.1 × 10^−6^ esu). The evaluated optical switching figures of merit met the requirements for all-optical switching and optical limiting. DFT and TDDFT calculations demonstrated that donor substitution and conjugation length govern electronic structure, charge transfer character, and global reactivity descriptors. Enhanced electronic softness and hyperpolarizability in Dye B further support its superior refractive nonlinearity. These results establish clear structure–property correlations and highlight donor engineering as an effective strategy for developing organic nonlinear optical and photonic materials.

## 1. Introduction

Advances in photonic and optoelectronic technologies are laying the foundation for the next generation of devices, and this development has placed advanced optical materials at the center of modern research. Materials with strong nonlinear optical (NLO) responses have recently attracted significant attention because of their broad range of applications, including optical switching, optical limiting, optical data storage, and biomedical imaging [1,2].

Among the broad classes of NLO materials (such as inorganic molecules and metal complexes), organic molecules have become attractive candidates due to their tunable donor–acceptor strength and π-conjugation length, which enables the development of high-performance chromophores with significant third-order nonlinear responses [3]. Organic molecules with a donor–π–acceptor (D-π-A) conjugation have been shown in several studies to possess a diverse range of optical, electrical, and optoelectronic properties [4,5]. Recently, amid the broad class of anthracene-based polycyclic aromatic ketone dyes, 7H-benz(de)anthracen-7-one derivatives have been widely recognized for their excellent luminescent properties, strong intramolecular charge transfer (ICT), and compatibility with donor–π–acceptor engineering [6,7]. Introducing the ethynyl (–C≡C–) between the benzanthrone core and the donor moiety effectively extends the conjugation pathways by promoting strong electronic transfer. This effect enhances conjugation control over third-order nonlinear properties. Despite this potential, a systematic investigation correlating substituent effects with experimental NLO responses in ethynyl-benzanthrone derivatives remains to be carried out.

The NLO behavior of an organic molecule is strongly influenced by the position of the substitution and donor–acceptor groups, and these factors collectively perturb the electron distribution along the π-conjugated framework [8,9,10,11]. Appropriately chosen electron-donating or electron-withdrawing groups can modulate the HOMO−LUMO energy separation, alter charge transfer character, and consequently affect both the real and imaginary components of third-order susceptibility (χ^3^). Among many experimental techniques, the Z-scan method is a reliable and widely used experimental technique that provides precise measurements of nonlinear parameters.

Density functional theory (DFT) and its time-dependent DFT (TDDFT) serve as powerful computational approaches for examining the electronic structures of molecules in both ground and excited states. These approaches enable the prediction of molecular hyperpolarizability, estimation of refractive indices, analysis of the frontier molecular orbitals, and visualization of charge transfer processes. However, the computational results provide critical insights into the origin of the observed nonlinear response and allow us to visualize the structural behavior of the molecule.

Although previous studies have reported the notable NLO properties of benzanthrone-based systems [7,12] to the best of our knowledge, there is a dearth of work on ethynyl-extended benzanthrones, where substituent effects are systematically varied and evaluated using range-separated hybrid density functional theory and Z-scan experiments. This leaves a clear gap in our understanding of how specific donor or acceptor groups alter charge transfer efficiency, dictate the sign and magnitude of nonlinear parameters, and influence the overall optical performance of ethynyl-extended-benzanthrone compounds.

**Figure 1:**
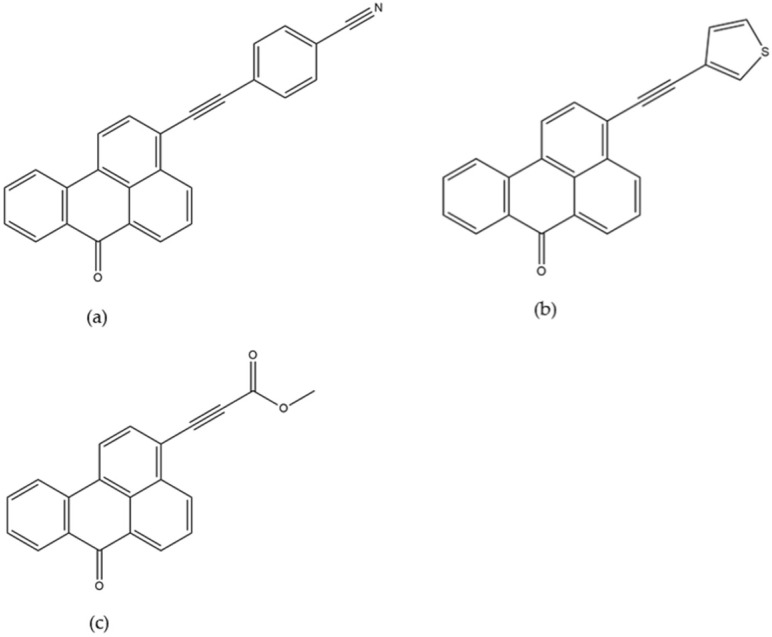
Molecular structures of Dyes A (**a**), B (**b**), and C (**c**).

Herein, we report the substituent effects of three newly synthesized ethynyl-benzanthrone derivatives—namely, 4-((7-oxo-7H-benzo[de]anthracen-3-yl)ethynyl)benzonitrile (Dye A), 3-(thiophen-3-ylethynyl)-7H-benzo[de]anthracen-7-one (Dye B), and methyl 3-(7-oxo-7H-benzo[de]anthracen-3-yl) propiolate (Dye C)—on their NLO behavior through both computational and experimental approaches. The structures of the dyes examined in this work are presented in Figure 1. The selection of these three substituents was designed to systematically probe different electronic environments within the benzanthrone framework. The benzonitrile group in Dye A acts as a strong electron-withdrawing substituent, promoting charge localization and enhancing ICT characteristics. The thiophene unit in Dye B introduces an electron-rich, π-conjugated system that facilitates charge delocalization, and the methyl propiolate group in Dye C provides comparatively weaker electron withdrawal and a more rigid electronic environment. This deliberate variation allows for a comparative investigation of how substituent-induced modulation of electronic structure influences nonlinear behavior.

## 2. Results and Discussion

### 2.1. Synthesis and Characterization

**Scheme 1:**
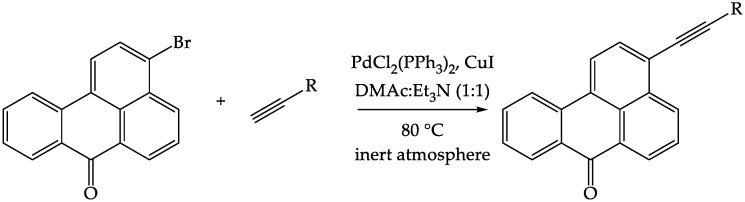
Synthetic route for the preparation of the studied dyes.

The Sonogashira coupling reaction was used for the synthesis of dyes under present investigation, which is often applied in preparative chemistry for the synthesis of alkynes [13]. The reaction involves the interaction of a terminal alkyne with a palladium catalyst, a copper (I) cocatalyst, and an aryl halide (see Scheme 1). The reaction proceeds in several steps, beginning with the oxidative addition of the palladium catalyst to the aryl halide or vinyl halide and ending with the reductive elimination of the desired alkyne product. The copper (I) cocatalyst plays a key role in facilitating the transmetallation step.

The identification of prepared substance’s structure was accomplished using IR, NMR spectra data, and HRMS data. The obtained spectroscopic data fully confirm the structures of the synthesized derivatives. The infrared spectra obtained show characteristic peaks corresponding to the benzanthrone carbonyl group (C=O) vibrations at 1650–1665 cm^−1^, as well as carbon–carbon triple-bond (C≡C) vibrations at around 2200–2230 cm^−1^, which are typically observed in alkynes.

The infrared spectra of the obtained compounds have the following characteristic absorption bands: 1592–1647 cm^−1^, which corresponds to a conjugated C = N bond; C = O (1640–1668 cm^−1^); and C = C (1512–1574 cm^−1^). There are also fluctuations of the C–H bond (2924–3073 cm^−1^). The 1H NMR spectra clearly show a typical multiplet signal of aromatic protons at 7.50–9.00 ppm, consisting of nine protons of the benzanthrone residue and, additionally, four protons of phenyl group (dye A) or three protons of the thiophene cycle (dye B). Detailed IR, NMR, and high-resolution mass spectroscopic data along with the spectra for all the compounds are provided in the supporting information.

**Figure 2:**
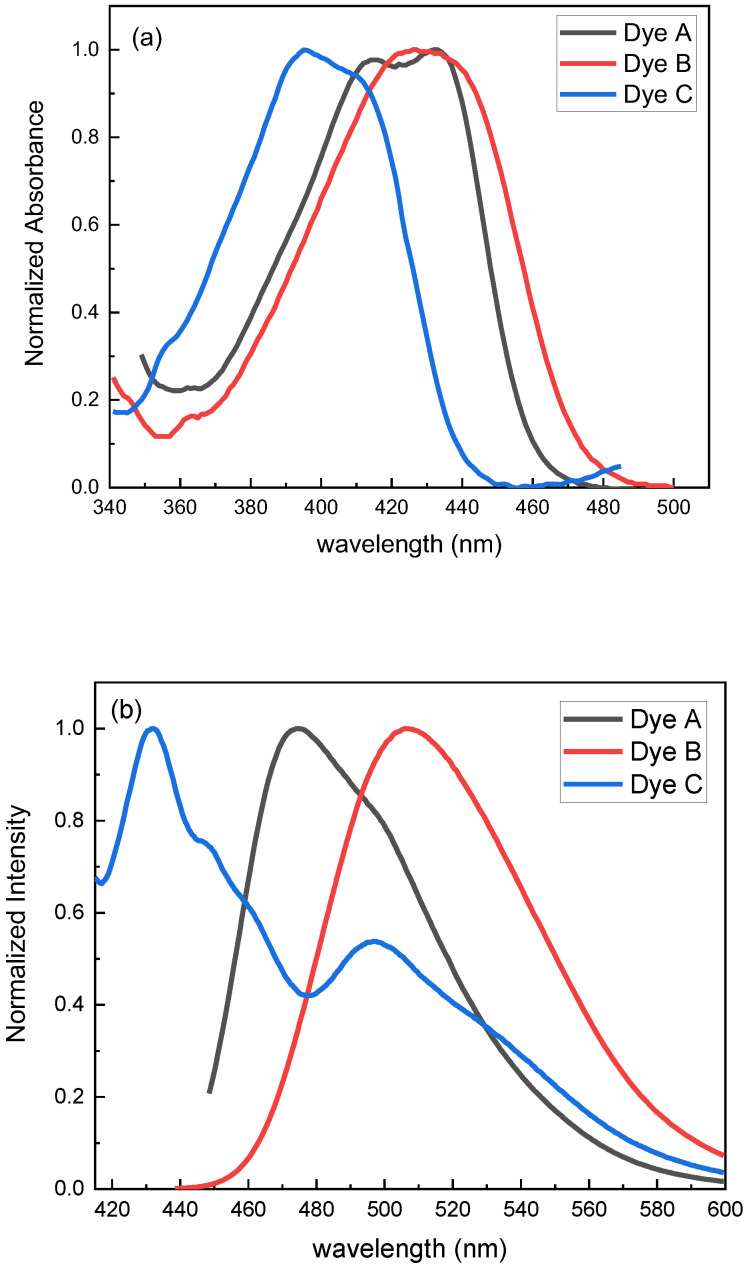
(**a**) Absorption spectra of dye A, B, and C. (**b**) Steady-state emission spectra of dye A, B, and C.

Absorption and steady-state emission spectra for all three dyes are shown in Figure 2a,b, respectively. Absorption maxima for dye A, B, and C were 433 nm, 427 nm, and 395 nm, respectively, and emission maxima were found to be 471 nm for dye A, 502.5 nm for dye B, and 432 nm for dye C. In the linear absorption studies for all three dyes recorded in DMF, no significant absorption band was observed in the 532 nm range. This indicates that the molecules did not undergo any strong one-photon electronic transition at this wavelength. Therefore, further insight into their optical behavior at 532 nm cannot be obtained from UV–visible data. To analyze the molecular response under an intense optical field, the Z-scan technique was employed.

### 2.2. Open-Aperture Z-Scan Technique

In the open-aperture (OA) configuration, the entire transmitted beam was collected by the detector to determine the nonlinear absorption coefficient (βeff). This can be analyzed without placing the aperture in front of the detector. This setup eliminates the effects due to refraction or phase distortion, obtaining nonlinear absorption processes such as reverse saturable absorption (RSA), saturable absorption (SA), and two-photon absorption (TPA). Therefore, it investigates how the absorption of a material changes with incident intensity.

The data obtained from open-aperture scanning is theoretically fitted using Equation (1) to calculate the βeff [14,15,16].

(1) $$T(Z)=1-\frac{\beta effI_{{}^{\circ}}L_{eff}}{2\surd 2(1+\frac{Z^{2}}{Z_{{}^{\circ}}^{2}})}$$

where T(Z) represents the transmittance at position Z, I_0_ is the on-axis peak intensity at focus (Z = 0), and *L_eff_* stands for the effective thickness of the sample. Z_0_ is the Rayleigh length, and it is calculated using the equation, $Z_{0}=\frac{\pi \omega_{0}}{\lambda }$. Here, $\omega_{0}$ and λ denote the beam waist and the wavelength of the light used, respectively.

The transition energies of three dyes were found to be 2.86 eV, 2.90 eV, 3.14 eV, respectively. The excitation photon energy (*hv*) used in the Z-scan experiment was 2.33 eV. However, the simultaneous absorption of two photons provided an effective excitation energy of 4.66 eV (2*hv*), which exceeded the lowest excited-state energies of all three dyes and allowed access to higher-lying electronic states (*hv_abs_* < 2*hv*). Therefore, the observed nonlinear absorption can be attributed predominantly to a two-photon absorption process.

**Figure 3:**
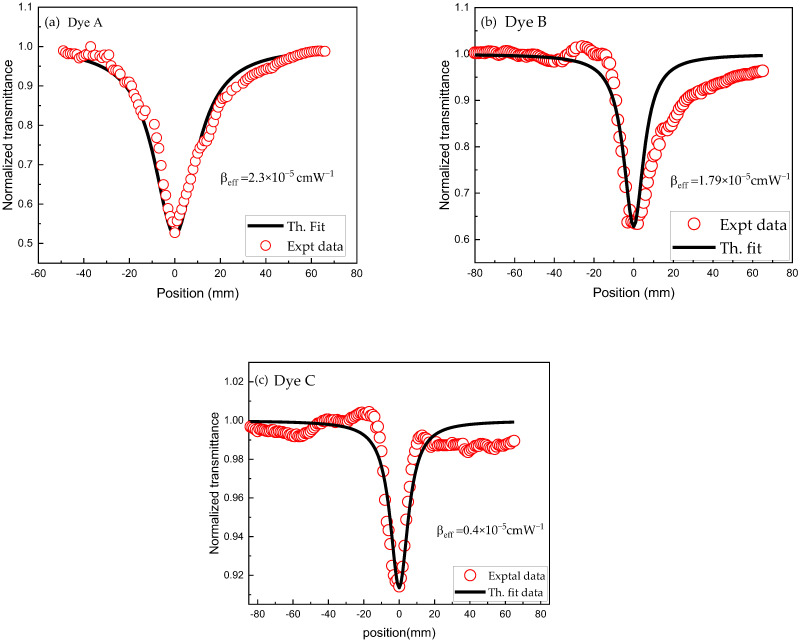
OA Z-scan measured for (**a**) dye A, (**b**) dye B, and (**c**) dye C samples. Solid curves are the theoretical fits to the experimental data.

The OA curves for dyes A, B, and C are shown in Figure 3a, b, and c, respectively. Here, all three figures manifest the symmetric valley centered at the focal point, revealing the behavior of RSA. This behavior arises from the increase in absorption with the laser intensity. For all three curves, the experimental data are well fitted with the standard two-photon absorption model (using Equation (1)). This indicates that the observed nonlinear absorption originates from simultaneous TPA. The βeff values of all three dyes are tabulated in Table 1. The nonlinear absorption coefficient was found to decrease with decreasing π-conjugation length among the three studied benzanthrone derivatives, i.e., βeff reaches a maximum in dye A (2.3 × 10^−5^ cm/W), and it decreases from dye A to dye C.

The two-photon absorption (2PA) cross-section, σ2PA = $hv\beta eff/NC\times 10^{-3}$, quantifies the efficiency of nonlinear excitation from the ground state via the simultaneous absorption of two photons. It is expressed in Göppert–Mayer (GM) units, where 1 GM = 10^−50^ cm^4^ s photon^−1^ molecule^−1^. The calculated 2PA cross-sections for dye A, dye B, and dye C are 1.2 × 10^6^, 1.0 × 10^6^, and 0.23 × 10^6^ GM, respectively. The benzonitrile group in dye A, being a strong electron acceptor, promotes the localized excited-state population and enhances absorption-dominated nonlinearities, resulting in higher βeff and σ2PA. In the case of dye B, although thiophene is an electron-donating substituent, the absorptive nonlinear parameters of dye B are lower than those of the benzonitrile substitution. This is attributed to the enhanced delocalization of charge in the thiophene system, which distributes the excited-state electron density over the conjugated framework rather than localizing it in discrete electronic states. Such delocalization reduces the probability of βeff and σ2PA. In propiolate-substituted dye C, absorptive nonlinearity further reduces due to the dual role of the propiolate group as a rigid π-spacer and moderate π-acceptor, which promotes refractive polarization rather than excited-state absorption. Therefore, dye C exhibits the lowest absorptive nonlinear response among all systems.

### 2.3. Closed-Aperture Z-Scan Technique

**Figure 4:**
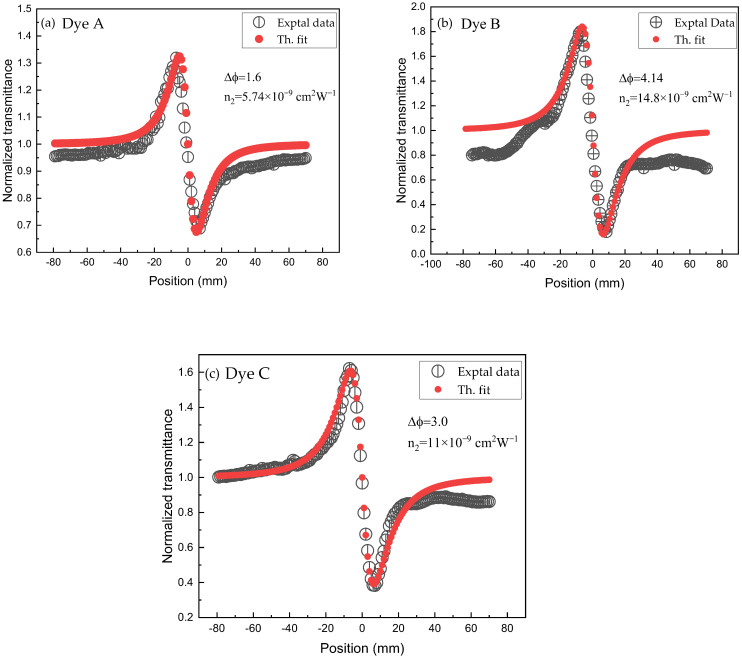
CA Z-scan measure for (**a**) dye A, (**b**) dye B, and (**c**) dye C. Red dotted lines are the theoretical fits to the experimental data.

In this measurement, only the on-axis part of the transmitted beam is collected through an aperture before the detector to determine the nonlinear refractive index (n_2_). The closed-aperture data from the z-scan analysis is fitted using Equation (2) [14,15].

(2) $$T(Z)=1-\frac{\left(4X∆\emptyset_{{}^{\circ}}\right)}{\left(X^{2}+1\right)(X^{2}+9)}$$

where X = Z/Z_O_, and $∆\emptyset_{{}^{\circ}}$ is the on-axis phase shift. n_2_ can be calculated using the following relation:

(3) $$n_{2}=\frac{∆\emptyset_{{}^{\circ}}}{kI_{{}^{\circ}}L_{eff}}$$

k is the propagation constant; it can be written as k = 2π/λ. CA curves for all three dyes A, B, and C are presented in Figure 4a, b, and c, respectively. The peak followed by the valley in all three CA graphs shows that n_2_ < 0, confirming the defocusing behavior of all three dyes.

The substitution of thiophene to the benzanthrone core results in heavier atoms, which influence electronic transitions and improve nonlinearity.

Here, the n_2_ is found to reach a maximum in dye B, and it is n_2_ = 14.8 × 10^−9^ cm^2^/W. $\chi^{\left(3\right)}$ is a complex quantity, and it can be calculated using both the real and imaginary part of the susceptibility from Equation (4), where the real part of susceptibility ($\chi_{R}^{\left(3\right)}$) arises from n_2_, and the imaginary part of susceptibility arises from βeff [14].

(4) $$\left|\chi^{\left(3\right)}\right|=\sqrt{{\left|\chi_{R}^{\left(3\right)}\right|}^{2}+{\left|\chi_{I}^{\left(3\right)}\right|}^{2}}$$

where

(5) $$\chi_{R}^{\left(3\right)}(esu)=\frac{cn_{{}^{\circ}}^{2}}{120\pi^{2}}n_{2}\;(m^{2}/W)$$

(6) $$\chi_{I}^{\left(3\right)}(esu)=\frac{c^{2}n_{{}^{\circ}}^{2}}{240\pi^{2}\omega }\beta eff\;(m/W)$$

The hyperpolarizability ($\gamma_{h}$) mainly depends on how easily electrons move by the application of an electric field, and it is calculated by the Equation (7). γ_h_ reaches a maximum in dye B, and it is found to be 3.84 × 10^−26^ esu.

(7) γh=χ(3)[13n°2+2]4N

The thiophene-substituted molecule (dye B) exhibits the highest hyperpolarizability because thiophene provides a more polarizable and softer electron donor compared to oxygen-based substituents [4,5], thereby enhancing the charge transfer characteristics. Further, all the NLO properties obtained for all three dyes as obtained from the above equations are presented in Table 1. For a meaningful comparison, previously reported organic nonlinear materials with structurally related π-conjugated frameworks and measured under comparable experimental conditions are selected. These systems represent typical donor–π–acceptor chromophores widely studied in recent years for nonlinear optical applications. A comparative analysis of the NLO parameters (Table 2) indicates that the present chromophores exhibit competitive performance relative to previously reported organic NLO materials. In particular, dye A shows strong nonlinear absorption behavior, while dye B demonstrates enhanced nonlinear refractive response. The key advantage of the present system lies in its tunable structure–property relationship, where systematic variation of substituents allows for controlled modulation of nonlinear absorption and refractive behavior. This provides a useful design strategy for tailoring optical responses in organic chromophores.

The compounds are suitable for all optical switching applications only if they satisfy the figure-of-merit (FOM) conditions. FOM (W and T) is calculated using Equations (8) and (9) [20].

(8) $$W=\frac{n_{2}I}{\alpha_{0}\lambda }$$

(9) $$T=\frac{\beta eff\lambda }{n_{2}}$$

For all three dyes in the present work, the values calculated for W are greater than 1, and T is extremely low (T << 1), satisfying the FOM condition. This combination makes the dyes highly suitable for Kerr-type all-optical switching, ultrafast phase modulation, and photonic waveguide applications.

### 2.4. Optical Limiting

**Figure 5:**
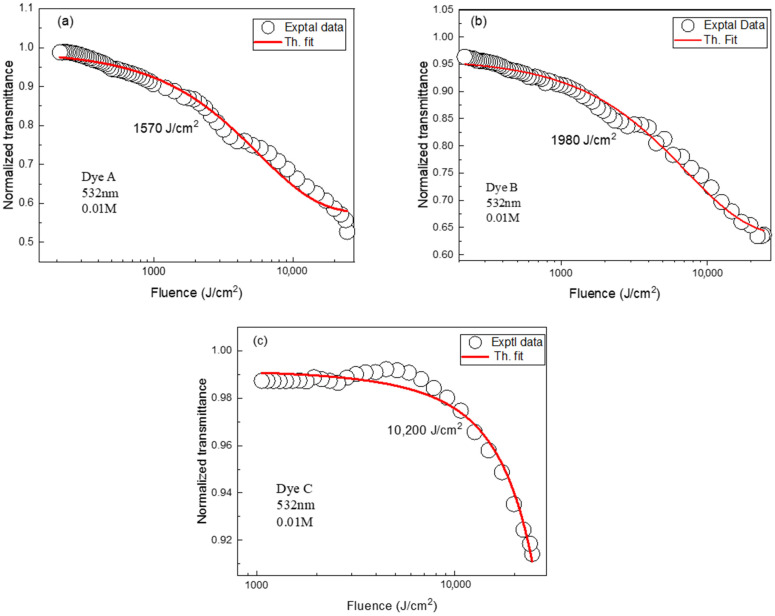
Optical limiting curves for (**a**) dye A, (**b**) dye B, and (**c**) dye C. Red solid lines represent the theoretical fit to the experimental data.

Optical limiting (OL) is an important nonlinear optical property in which a material exhibits high transmission at low incident light intensities while effectively attenuating high-intensity light, which protects the sensitive optical components, the human eye from laser-induced damage, and sensors [21,22]. A suitable optical limiting material is characterized by its ability to reduce the transmitted intensity beyond a certain input fluence, commonly referred to as the limiting threshold [23]. The optical limiting performance is often governed by nonlinear optical processes such as TPA and RSA. In this study, the optical limiting behavior of the three dyes was analyzed using OA Z-scan data. The normalized transmittance was plotted as a function of input fluence, as shown in Figure 5. All three optical limiting curves reveal a gradual decrease in transmittance with increasing input fluence, followed by a deviation from linearity beyond a certain fluence range. Based on this analysis, the optical limiting threshold values are estimated to be approximately 1.57 kJ/cm^2^, 1.98 kJ/cm^2^, and 10.2 kJ/cm^2^ for dye A, dye B, dye C, respectively. These high threshold values indicate that nonlinear attenuation occurs only at high incident fluence, suggesting that the observed optical limiting behavior is associated with late-onset nonlinear processes rather than low-threshold protective limiting. To further understand the origin of the observed nonlinear optical behavior, theoretical calculations were carried out.

### 2.5. Computational Calculation of NLO Parameters

To establish the clear connection between theoretical calculations and the experimentally observed NLO behavior, the following discussion focuses on key electronic parameters that govern NLO response. It should be noted that the calculated hyperpolarizability values represent intrinsic molecular properties, whereas the experimentally measured NLO parameters correspond to effective macroscopic responses. Therefore, direct quantitative comparison is not appropriate; however, qualitative trends can be meaningfully correlated. The first hyperpolarizability tensor β plays a crucial role in understanding the second-order nonlinear optical properties of NLO active molecules. The first hyperpolarizability (β) value for all three designed benzanthrone derivatives was calculated using the LC-BLYP functional with CPKS (coupled–perturbed Kohn–Sham) response formalism in the gas phase, and the values are presented in Table S1. The results include static β and frequency-dependent β for electro-optical Pockels effect (EOPE; β(−ω, ω, 0)) and second harmonic generation (SHG; β(−2ω, ω, ω)) at 532 nm wavelength. All β values are in 10^−30^ esu. The static hyperpolarizability of the benzanthrone with benzonitrile substitution is 8.23, and for propiolate substitution it is 6.796. Similarly, the thiophene donor extension on benzanthrone increases the β(0) by ~2 times, resulting in the value of 13.77, which indicates that the substitution of the donor enhances the β. The frequency-dependent β values for the electro-optical Pockels effect (EOPE) and second-harmonic generation (SHG) were also investigated at a wavelength of 532 nm. The estimated dynamic hyperpolarizabilities β(ω) are higher than the static hyperpolarizability β(0), and the result shows a similar trend, with the thiophene substituent exhibiting higher β(ω) than the benzonitrile and propiolate substituent. This trend is consistent with the experimental observations, where dye B exhibits enhances NLO response, indicating that increased π-electron delocalization plays a key role in governing the observed behavior.

The second-order hyperpolarizability (γ) reflects the third-order nonlinear response originating from higher-order electronic polarization under both static and oscillating electric fields. In static hyperpolarizability (γ(0)), dye A shows the maximum response. This behavior can be associated with the strong ground-state polarity introduced by the nitrile substituent. The electron-withdrawing nature of the cyano group increases the longitudinal charge separation in the conjugated framework. This leads to an increase in the intrinsic polarization and the static γ value. The experimentally obtained nonlinear refractive index corresponds to the response under the dynamic optical field at 532 nm. In the frequency-dependent regime, nonlinear response becomes strongly influenced by π-electron delocalization. Under these conditions, dye B shows a stronger dynamic γ contribution, which is consistent with the extended conjugation and higher polarizability produced by the substituent. These results indicate that static and dynamic third-order responses are governed by different properties of the electronic structure. While static γ is largely determined by ground-state polarity and permanent charge separation, the dynamic nonlinear response depends more strongly on electronic flexibility and conjugative delocalization under an applied optical field. The consistency between the calculated dynamic γ trends and the measured refractive nonlinearities suggests that, in these systems, refractive nonlinearity is primarily controlled by substituent-induced modulation of electronic softness rather than by dipole strength alone. Overall, the theoretical results provide a consistent explanation of the experimental trends; dye A, with stronger charge localization, favors absorption-dominated nonlinear behavior, while dye B, with enhanced π-conjugation and electronic softness, exhibits stronger refractive nonlinear response. Dye C, characterized by comparatively higher rigidity, shows weaker nonlinear activity. The calculated static and dynamic second-order hyperpolarizabilities in the gas phase for all the dyes are listed in Table S2. The ground-state dipole moments were calculated at optimized geometries to assess substituent-induced polarity within the benzanthrone framework. The computed dipole moments follow the order dye C (4.18 D), then dye A (4.11 D) and dye B (3.31 D), indicating the stronger permanent charge separation in benzonitrile- and propiolate-substituted systems. Two polarizability parameters are of particular relevance: the isotropic (average) polarizability ⟨α⟩, and the anisotropic polarizability (Δα). These quantities are evaluated from the Cartesian components (αij) of the polarizability tensor using the following relations [24,25,26].

(10) $$\left\langle \alpha \rangle =\frac{1}{3}\;[\alpha xx+\alpha yy+\alpha zz\right]$$

(11) $$\Delta \alpha =\frac{1}{\sqrt{2}}\;\{[(\alpha xx-\alpha yy)2+(\alpha yy-\alpha zz)2+(\alpha zz-\alpha xx)2]+6(\alpha_{xy}^{2}+\alpha_{xz}^{2}+\alpha_{yz}^{2})\}1/2$$

The refractive indices (n) of the three dyes in DMF were also evaluated using the Lorentz–Lorenz Equation (12):

(12) $$\frac{n^{2}-1}{n^{2}+2}=\frac{4\pi \langle \alpha \rangle }{3V_{mol}}$$

where V_mol_ denotes the molecular volume. The molecular volumes were calculated at the LC-B3LYP/6-311G(d,p) level of theory to achieve higher computational accuracy. All the calculated values are tabulated in Table S3.

### 2.6. Frontier Molecular Orbital Analysis

The highest occupied molecular orbital (HOMO) and lowest unoccupied molecular orbital (LUMO) energy separation is a crucial qualitative indicator of electronic stability and reactivity in the frontier molecular orbital framework. Greater gaps result in less orbital mixing and a decreased likelihood of charge transfer interactions [27]. On this basis, a comparative analysis of the benzonitrile-, thiophene-, and methyl propiolate-substituted benzanthrone derivatives shows the clear substituent-driven modulation of the electronic structure under identical solvent conditions. The frontier orbitals were obtained from ground-state DFT calculations at the optimized geometry. Across all three dyes, the HOMO is predominantly delocalized over the benzanthrone π-conjugated core, with the electron-donating character largely confined to the aromatic framework. The LUMO also spreads along the conjugated backbone, although its distribution and energy are shaped by the nature of the substituent. For the benzonitrile-substituted derivative (dye A), the electron-withdrawing cyano group stabilizes the LUMO more strongly, which narrows the HOMO−LUMO gap to about 6.45 eV in DMF.

For the thiophene-substituted molecule (dye B), the substitution ring extends the π-conjugation through efficient orbital overlap with the benzanthrone core. This orbital overlap leads to a slight decrease in the energy gap in the solvent as compared to the gas phase. The LUMO shows the increased extension near the thiophene substitution, which leads to an enhancement of conjugative delocalization rather than strong electron-withdrawing stabilization. From this observation, we can say that the dye B maintains efficient electronic communication across the π-conjugated pathway. With methyl propiolate substitution (dye C), the presence of an ester-functionalized alkynyl moiety with strong electron-withdrawing character leads to the stabilization of the occupied manifold, resulting in an increase in the HOMO−LUMO gap among the three dyes (~6.7 eV in gas phase). The larger the orbital separation, the greater the electronic hardness and the weaker the orbital mixing; this reduces the likelihood of the charge transfer process. Although the HOMO−LUMO energy gaps in all three dyes vary slightly, even the very small variations in the frontier orbital stabilization affect how each system responds under optical excitation. In dye A, the LUMO is stabilized by the nitrile substitution, which increases the acceptor character and supports the excited-state population; it is partially responsible for the stronger absorptive nonlinearity. These observations reveal that the nonlinear behavior not only depends on the HOMO−LUMO gap but also depends on the balance between the orbital delocalization, substituent-induced stabilization, and electronic softness.

**Figure 6:**
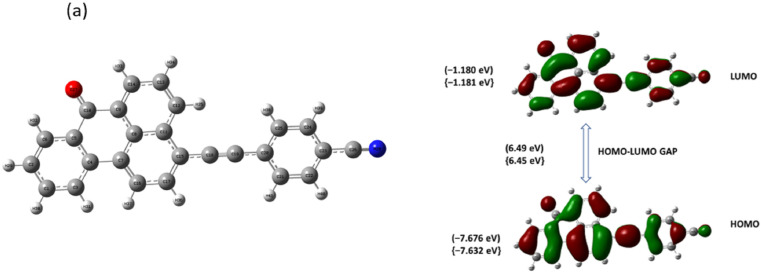

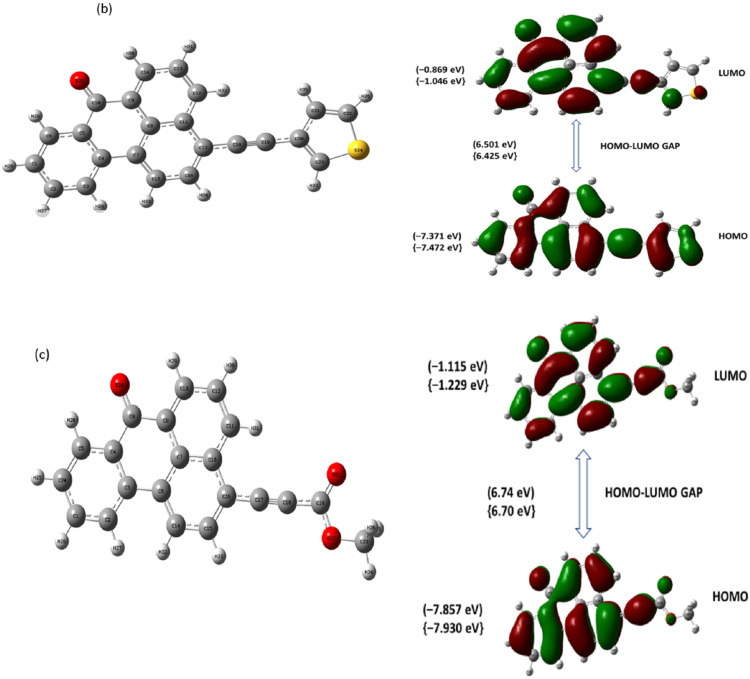
Optimized structure and frontier molecular orbit of (**a**) dye A, (**b**) dye B, and (**c**) dye C; values inside ( ) and { } are in the gas phase and DMF, respectively.

The optimized geometries along with the corresponding HOMO−LUMO distributions and calculated energy gaps for dyes A–C are presented in Figure 6a–c.

### 2.7. Electron Density Difference Analysis

**Figure 7:**
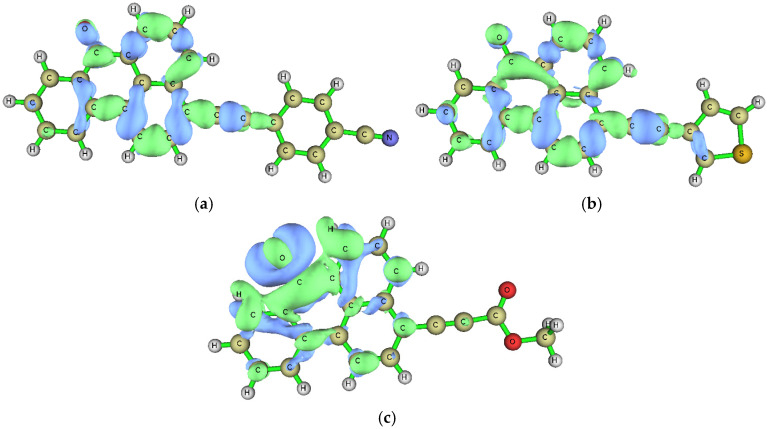
EDD map of dye A (**a**), dye B (**b**), and dye C (**c**).

To understand the nature of excitation-induced electronic reorganization, electron density difference (EDD) analysis was carried out by subtracting the ground-state electron density from that of the first singlet excited state, as shown in Figure 7a–c. In EDD maps, the green (ρ+) and blue (ρ-) regions correspond to electron density accumulation and depletion, respectively, following excitation. For all three dyes, electron depletion is primarily distributed over the benzanthrone, while electron accumulation (ρ^+^) extends along the conjugated backbone, with the extent of redistribution modulated by the nature of the substituent. Dye A exhibits the smallest centroid separation (D = 0.25 Å) and the most negative t-index (−1.67 Å), along with near-unity electron–hole overlap. These values indicate strong spatial co-localization of electron and hole densities, which confirms that the excitation is predominantly localized in nature. The small centroid displacement indicates that photoexcitation mainly involves redistribution of electrons within the aromatic core rather than a directional movement along the molecular backbone. Dye B shows the largest centroid separation (D = 1.34 Å) and a moderately negative t-index (−1.38 Å), which indicates an increased spatial displacement between electron and hole centroids. Although the separation remains within the short-range regime, the greater displacement compared to dye A indicates more excitation-induced polarization along the conjugated pathway. The substituent, therefore, increases the partial directional charge redistribution while preserving substantial orbital overlap. Dye C has an intermediate centroid separation (D = 0.83 Å) with a less negative t-index (−1.01 Å) among the three dyes. This combination suggests stronger electronic reorganization relative to dyes A and B, which is characterized by increased polarization within the π-framework. However, the centroid separation remains in sub-angstroms, which indicates that the excitation does not evolve into a long-range charge transfer state but remains confined within the conjugated framework. These findings align with the frontier orbital analysis and confirm that excitation in these ethynyl-extended benzanthrone derivatives continues to be of a largely delocalized π–π* character.

### 2.8. Substituent-Dependent Global Reactivity Descriptors

To further quantify the substituent-dependent electronic characteristics, global reactivity descriptors, such as global hardness (*η*), chemical potential (μ), softness (S), electronegativity (χ), and electrophilicity (ω), are calculated using the following equations [28]:

(13) $$\eta ={(E}_{L}-E_{H})/2$$

(14) $$\mu =(E_{L}+E_{H})/2$$

(15) $$S=\frac{1}{2\eta }$$

(16) $$\omega =\frac{1}{4}\frac{{\left(E_{L}+E_{H}\right)}^{2}}{\left(E_{L}-E_{H}\right)}$$

In these expressions, E_H_ and E_L_ correspond to the energies of the HOMO and LUMO, respectively. As summarized in Table 2, thiophene-substituted dye B shows the strongest nonlinear optical response among the three ethynyl extended dyes, particularly in terms of refractive nonlinearity and overall third-order susceptibility. This increase in the nonlinearity in dye B originates from the lower electrophilic and less negative chemical potential (μ). These two properties of the molecule increase the electron donation and π-electron delocalization when the electric field is applied externally. Here, the larger electronic softness and the extended π-conjugation are also responsible for efficient ICT, increasing the nonlinear polarization. Dye A exhibits intermediate values of chemical hardness and softness, indicating a balanced electronic response with moderate flexibility towards charge redistribution. Its chemical potential reflects a stabilized electronic structure with appreciable electron-accepting capacity. Dye B shows similar chemical hardness to dye A and similar softness; however, its lower negative chemical potential (μ = −4.12eV) suggests a relatively higher tendency to donate electron density, consistent with the electron-rich and π-conjugated nature of the thiophene substituent. In contrast, dye C stands out for its highest chemical hardness and lowest softness, indicative of increased electronic rigidity imposed by the strongly electron-withdrawing propiolate group. The combination of a highly negative chemical potential and the largest electronegativity and elevated electrophilicity index further underscores the pronounced electron-accepting character and enhanced electronic stability of dye C. Dye C’s NLO behaviour depends mainly on electronic stabilization rather than polarizability, which results in reduced nonlinear efficiency.

In Table 3, *E*_(_*_H_*_−1)_ and *E*_(*L*+1)_ denote the energies of the HOMO−1 and LUMO+1 orbitals, respectively. ΔE_H−L_ and ΔE_(H−1)−(L+1)_ represent the energy gaps between HOMO−LUMO and HOMO−1−LUMO+1 orbitals, respectively.

### 2.9. Molecular Electrostatic Potential Studies

**Figure 8:**
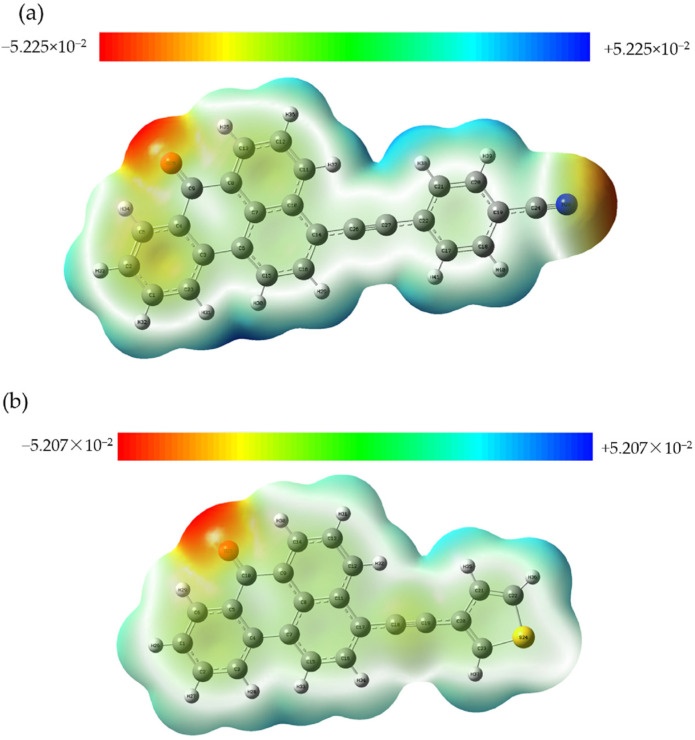

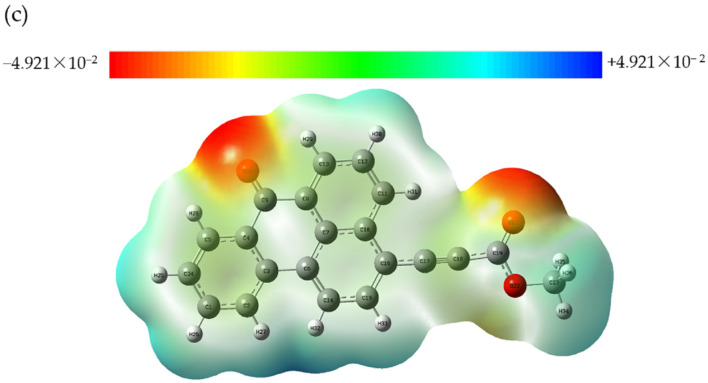
Molecular electrostatic potential map of dye A (**a**), dye B (**b**), and dye C (**c**).

The molecular electrostatic potential (MEP) analysis was performed to study how charge is distributed across the substituted dye molecules and to identify regions that are more likely to participate in local reactive interaction [29,30]. The MEP surfaces were generated using DFT-optimized geometries and are illustrated in Figure 8. In these representations, the electrostatic potential is mapped onto the molecular surface using a continuous color scale ranging from red to blue, where red denotes regions of high electron density (negative potential), blue represents electron-deficient regions (positive potential), and intermediate colors such as green and yellow correspond to moderate electrostatic potential values. The benzonitrile-substituted system exhibits the widest MEP range (−5.225 × 10^−2^ to +5.225 × 10^−2^ a.u.). For dye A, the MEP surface shows clear electron-rich regions (red to orange) that are localized around the nitrile nitrogen atom, indicating a strong negative electrostatic potential and highlighting this site as a favorable region for electrophilic interactions. The aromatic π-system exhibits predominantly green to yellow coloration, suggesting moderate charge delocalization across the conjugated framework. Electron-deficient regions (blue) are mainly distributed over hydrogen atoms, reflecting their electropositive nature. In the thiophene-substituted molecule, the highest negative electrostatic potential is concentrated around the sulfur atom of the thiophene ring, confirming its role as a potential nucleophilic site. The conjugated backbone displays relatively uniform green coloration, indicative of effective π-electron delocalization throughout the molecular framework. Compared to dye A, the charge distribution is more evenly spread, consistent with the less polar and more conjugative nature of the thiophene substituent. Electropositive regions remain localized around hydrogen atoms. For the dye C, strong negative potential regions are distinctly observed around the carbonyl oxygen atoms of the ester group, identifying them as the most reactive nucleophilic centers. A secondary electron-rich region is also visible along the alkynyl linkage, reflecting conjugation with the carbonyl functionality. The remainder of the molecular surface exhibits moderate electrostatic potential, while positive potential regions are again associated with hydrogen atoms.

## 3. Materials and Methods

### 3.1. General

All chemicals and solvents used were of analytical grade purchased from Sigma Aldrich (Munich, Germany), and were used as received. The course of the reactions and the preliminary evaluation of product purity were examined using thin-layer chromatography (TLC), carried out on MERCK Silica gel F254 plates (Darmstadt, Germany). A mixture of hexane, chloroform, and acetone (4:2:1, *v*/*v*/*v*) served as the developing solvent, and the chromatograms were visualized under ultraviolet illumination. Melting point measurements were performed using an MP70 Melting Point System (Mettler-Toledo, Greifensee, Switzerland), and the obtained values are presented without correction. The nuclear magnetic resonance analyses (^1^H- and ^13^C-NMR) of the molecules were recorded at room temperature on a Bruker Avance 500 MHz spectrometer (Billerica, MA, USA), and residual solvent signals were used for internal calibration. Infrared spectra were obtained employing a Perkin–Elmer Spectrum BX FTIR instrument (Waltham, MA, USA), using KBr pellets, across a spectral window of 4000–450 cm^−1^. High-resolution mass spectra were collected using an Agilent 1290 Infinity UPLC (Santa Clara, CA, USA) coupled with an Agilent 6230 TOF mass spectrometer. General synthesis methodology and structural information are provided in the supporting information.

All three dyes were subjected to UV–visible absorption and steady-state fluorescence studies in N-N-dimethylformamide (DMF) to characterize their linear optical behavior. The UV–visible absorption spectra were recorded using a LABINDIA spectrometer (Thane, India, model UV-3092), and the steady-state fluorescence spectra were recorded using a HITACHI (F-2700) spectrofluorometer (Tokyo, Japan). The concentration of the solutions for steady-state measurements was kept low to avoid an inner filter effect with optical density less than 0.1.

### 3.2. Z-Scan Technique

The Z-scan technique was used to investigate the third-order nonlinear optical properties of three ethynyl extended benzanthrone derivatives, denoted as dye A, dye B, and dye C. The measurements were performed on dilute solutions prepared in HPLC-grade DMF obtained from S.D. Fine-Chem Ltd. (Mumbai, India), selected for its high purity, optical transparency, and optical linearity in the visible region. The nonlinear optical measurements were performed using a continuous-wave (CW) diode-pumped solid-state (DPSS) laser (Holmarc instrument, Kochi, India) operating at 532 nm wavelength. The laser beam was focused using a convex lens of focal length 286 mm, yielding a beam waist of ω0 = 32.2 μm at a focal point, and the corresponding Rayleigh length Z0 = 6.15 mm was much greater than the effective thickness of the sample (Leff = 0.97 mm), confirming that the thin-sample condition (Leff << Z0) was satisfied. A 1 mm quartz cuvette with 0.01 M concentration was placed on a motorized linear translation stage, allowing controlled movement along the propagation axis (z). To detect the transmitted signal, a silicon photodiode was used. Pure DMF was scanned under identical conditions to confirm its negligible nonlinear response at 532 nm.

### 3.3. Quantum Chemical Calculations

The third-order nonlinear parameters of all dyes A-C were calculated computationally using the long-range corrected LC-BLYP functional with the 6-311+G(d,p) basis set. This included full Hartree–Fock exchange in the long-range region, which enables accurate NLO calculation in π-conjugated systems [31,32,33,34,35,36]. The ωB97XD/6-31G(d,p) level is widely employed for geometry optimization and frontier molecular orbital analysis of π-conjugated organic chromophores and benzanthrone derivatives [7,37,38]. Solvent-dependent calculations were incorporated using the IEFPCM solvation model, which allows consistent evaluation of the molecule’s behavior in DMF solvent [7,39,40]. Different density functionals were employed for specific computational tasks to ensure optimal accuracy. Geometry optimization and frontier molecular orbital analysis were carried out using the ωB97XD functional due to its reliable treatment of dispersion interactions, while the LC-BLYP functional was used for nonlinear optical property calculations because long-range corrected functionals provide a more accurate description of charge transfer interactions and hyperpolarizability in π-conjugated systems. All computational calculations mentioned above were performed using Gaussian 09 electronic structure simulation software [41]. The charge transfer characteristics were analysed using the Multiwfn wavefunction analysis software package (Version 3.7) [42].

## 4. Conclusions

In summary, the nonlinear optical behavior of the ethynyl extended benzanthrone derivatives was found to be strongly dependent on the nature of donor–acceptor substitution and the extent of π-conjugation within the molecular framework. Z-scan measurements clearly confirmed the reverse saturable absorption dominated by two-photon absorption and a significant nonlinear refractive response, with dye A exhibiting the highest nonlinear absorption and dye C exhibiting the lowest nonlinear absorption. Dye B showed the strongest nonlinear refraction. These experimental trends are well supported by theoretical insights, where HOMO−LUMO energy gaps rationalize substituent-dependent electronic stability, and molecular electrostatic potential maps reveal distinct charge localization and preferred interaction sites. Moreover, second-order hyperpolarizability calculations revealed pronounced static and dynamic third-order polarization, highlighting the role of molecular polarity and conjugative pathways in enhancing nonlinear response. Overall, these results establish effective structure–property relationships and demonstrate that rational donor–acceptor engineering in benzanthrone derivatives is a viable strategy for tuning nonlinear optical properties for photonic and optoelectronic applications.

## Figures and Tables

**Table 1:** Third-order nonlinear optical parameters of dye A, B, and C.

	β_eff_ (10^−5^ cm/W)	n_2_ (10^−9^ cm^2^/W)	Re χ^3^ (10^−6^ esu)	Im χ^3^ (10^−10^ esu)	χ^3^ (10^−6^ esu)	σ2PA (GM) 10^6^	γ_h_ (10^−26^ esu)	W	T (10^−2^)
Dye A	2.30	5.74	1.19	5.08	1.19	1.2	5.94	3.78	0.5
Dye B	1.79	14.8	3.10	3.96	3.10	1.0	15.37	9.77	0.2
Dye C	0.40	11.0	2.30	0.89	2.30	0.23	11.12	7.39	0.05

**Table 2:** Recently reported β_eff_ and n_2_ values of different materials with CW laser excitation.

Materials	β_eff_ (cm/W)	n_2_ (cm^2^/W)	Ref
Organic dye Nile blue	1.35 × 10^−5^	4.2 × 10^−9^	[17]
Fast green FCF dye	6.5 × 10^−5^	32 × 10^−9^	[18]
F3BC	5.5 × 10^−5^	4.5 × 10^−9^	[19]
F3NC	1.2 × 10^−5^	13.1 × 10^−9^	[19]
3-(4-Methylpiperazin-1-yl)-7Hbenzo[de]anthracen-7-one	10.9 × 10^−4^	9.03 × 10^−9^	[7]
3-(4-Phenylpiperazin-1-yl)-7H-benzo[de]anthracen-7-one	1.84 × 10^−4^	6.79 × 10^−9^	[7]

**Table 3:** Computationally calculated reactivity parameters of dye A, B, and C in DMF solvent.

Molecule	Dye A	Dye B	Dye C
E_H_ (eV)	−7.6325	−7.3710	−7.9307
E_L_ (eV)	−1.1812	−0.8696	−1.2294
E_(H−1)_ (eV)	−8.9925	−8.7187	−9.1800
E_(L+1)_ (eV)	−0.1659	0.3883	0.1458
∆E_H−L_ (eV)	6.4513	6.5014	6.7013
∆E_(H−1)−(L+1)_ (eV)	8.8266	9.1070	9.3258
*η* (eV)	3.2256	3.2507	3.3506
μ (eV)	−4.4068	−4.1203	−4.5800
S (1/eV)	0.1550	0.1538	0.1492
χ	4.4068	4.1203	4.5800
ω (eV)	3.0102	2.6112	3.1302

## Data Availability

The data presented in this study are available in this article and the Supplementary File.

## Supplementary Materials

The following supporting information can be downloaded at: https://www.mdpi.com/article/10.3390/molecules31091467/s1, NMR, IR, HRMS spectra.
