# Supplementary material for: Elucidating the Structure–Nonlinear Optical Property Relationship of Ethynyl Extended Benzanthrone Chromophores
Source: Molecules. 2026 Apr 28;31(9):1467. doi: 10.3390/molecules31091467 (PMC13164745; doi:10.3390/molecules31091467)
Supplement: Supplementary file 1 [file molecules-31-01467-s001.zip › molecules-4246097-supplementary.pdf]

Supporting information:

Elucidating the structure-nonlinear optical property relationship of ethynyl extended benzanthrone chromophores

Divya Jattu Gouda[1], B. Siddlingeshwar[1*], HM Suresh Kumar[2], Shivaraj R Maidur[3], S. R. Manohara[2], Armands Maleckis[4], Elena M. Kirilova[4*]

[1]*Department of Physics, M.S. Ramaiah Institute of Technology, (Autonomous Institute affiliated to VTU) Bengaluru-560054, India*

[2]*Department of Physics, Siddaganga Institute of Technology, (Autonomous Institute affiliated to VTU) Tumakuru-572103, India*

[3]*Department of Physics, Kristu Jayanti College, (Autonomous), Bengaluru-560077, India*

[4]*Department of Chemistry, Daugavpils University, Vienibas 13, Daugavpils, Latvia*

[*]*Correspondence to: B. Siddlingeshwar, E-mail: siddupysics@gmail.com*
Elena M Kirilova, E-mail: elena.kirilova@inbox.lv

Synthesis of novel dyes

General procedure:

3-Bromobenzanthrone (618 mg, 2 mmol), $PdCl_2(PPh_3)_2$ (14 mg, 0.01 mmol), CuI (4 mg, 0.01 mmol) and an acetylene derivative (2.4 mmol) are placed in a 50 mL round-bottom flask under an argon atmosphere followed by the addition of DMAc (10 mL) and Et_3N (10 mL). The reaction mixture is placed on a preheated oil bath at 80 °C and kept at this temperature for 0.5 – 1 h (TLC control). After completion, to the reaction mixture 5 mL of methanol is added. The resulting precipitate is filtered, thoroughly washed with 10 – 20 mL of methanol and dried. Recrystallized from chlorobenzene.

(Dye A): 3-((4-Cyanophenyl)ethynyl)-7H-benzo[de]anthracen-7-one

SMILES strings:

O=C1C2=CC=CC=C2C3=CC=C(C#CC4=CC=C(C#N)C=C4)C5=CC=CC1=C35

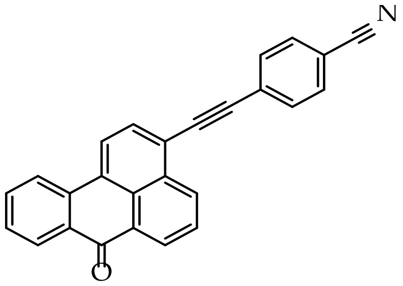

Bright orange solid. Yield: 71 %. Melting point: 273 °C. R_f = 0.54 (hexane/chloroform/acetone; 4:2:1). IR, λ_{max} (KBr) cm^{-1}: 1654 (C=O), 2226 (C≡C). ^1H NMR (500 MHz, Acetone-d_6) δ 8.85 (d, J = 8.3 Hz, 1H), 8.66 (t, J = 6.4 Hz, 2H), 8.52 (d, J = 8.1 Hz, 1H), 8.32 (d, J = 7.8 Hz, 1H), 8.01 (d, J = 7.7 Hz, 1H), 7.92 (t, J = 7.8 Hz, 1H), 7.86 (d, J = 8.0 Hz, 2H), 7.80 (d, J = 8.1 Hz, 2H), 7.77 (t, J = 7.6 Hz, 1H), 7.57 (t, J = 7.5 Hz, 1H). HRMS (ESI): m/z calculated for [$C_{26}H_{13}NO + H^+$] 356.1070, found 356.1070.

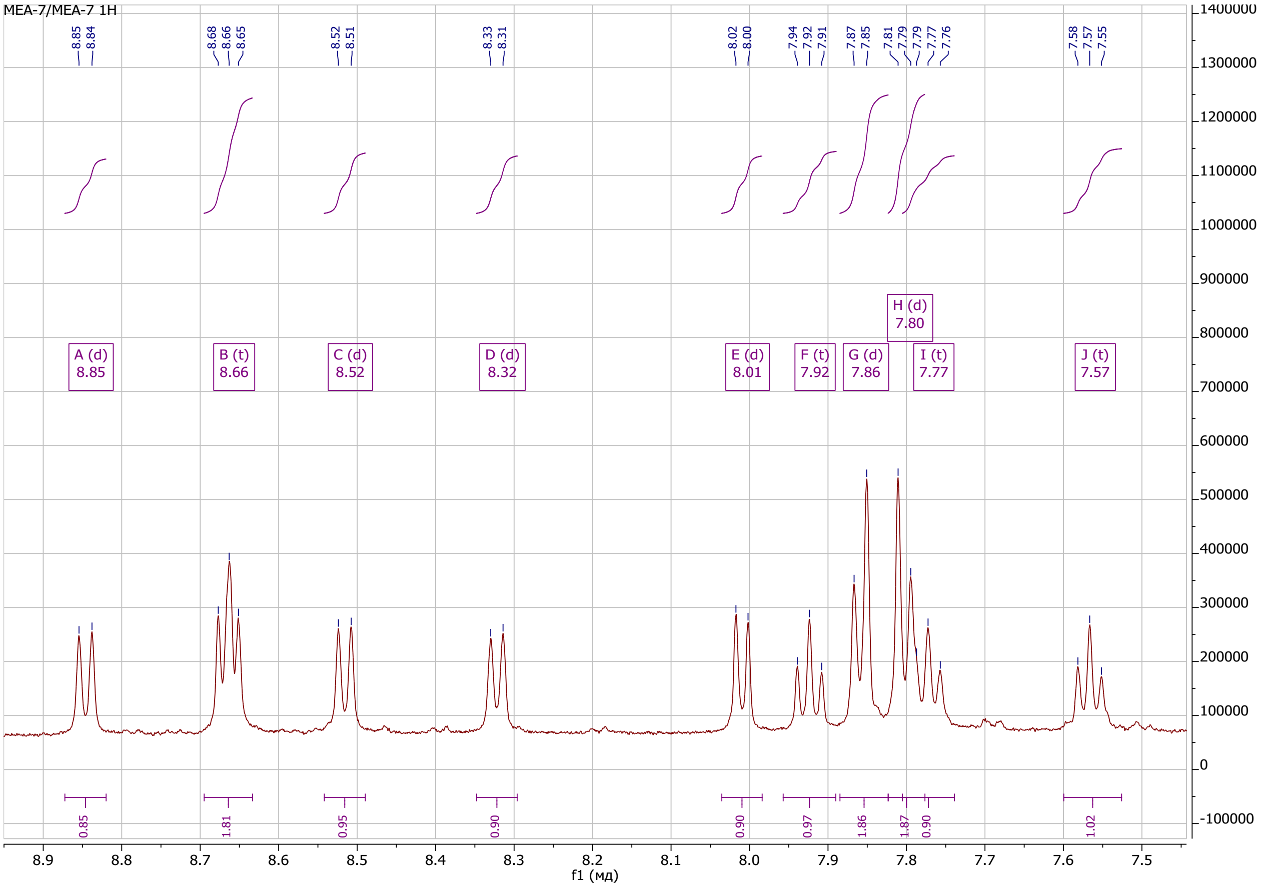

Fig. S1. Dye A: ^{1}H NMR spectrum; aromatic region (126 MHz, Acetone-d_6).

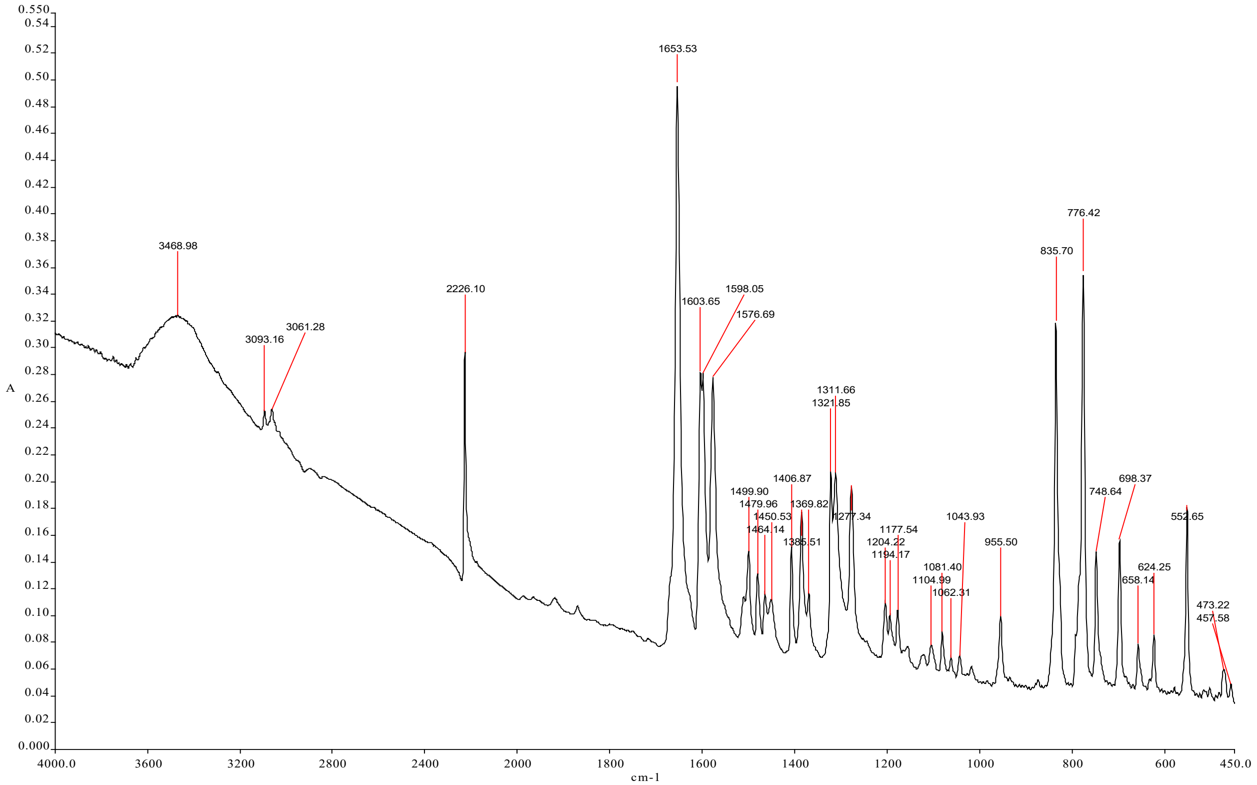

Fig. S2. Dye A: IR spectrum.

$[M+H]^+$ calc	$[M+H]^+$ found	Δ
356,1070	356,1070	0,0000

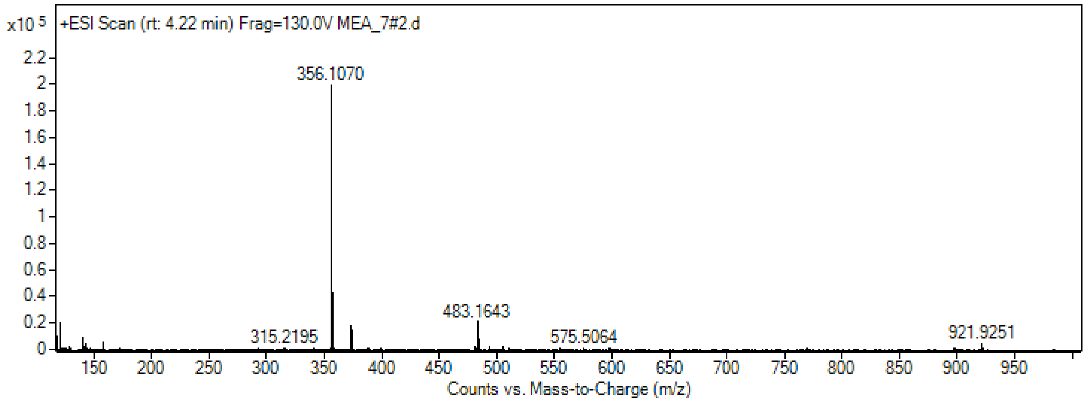

Fig. S3. High-resolution mass spectrum of Dye A.

(Dye B): 3-(Thiophen-3-ylethynyl)-7H-benzo[de]anthracen-7-one

SMILES strings: O=C1C2=CC=CC=C2C3=CC=C(C#CC4=CSC=C4)C5=CC=CC1=C35

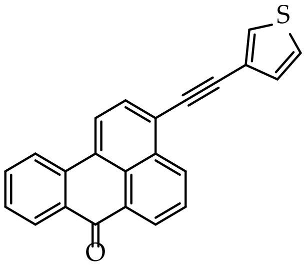

Yellow solid. Yield: 63 %. Melting point: 189 °C. R_f = 0.69 (hexane/chloroform/acetone; 4:2:1). IR, λ_{max} (KBr) cm^{-1}: 1663 (C=O), 2204 (C≡C). ^{1}H NMR (500 MHz, DMSO-d_6) δ 8.84 (d, J = 8.2 Hz, 1H), 8.75 (d, J = 7.9 Hz, 1H), 8.69 (d, J = 7.3 Hz, 1H), 8.62 (d, J = 8.2 Hz, 1H), 8.35 (d, J = 7.8 Hz, 1H), 8.15 (s, 1H), 8.03 (t, J = 7.9 Hz, 2H), 7.88 (t, J = 7.6 Hz, 1H), 7.75 (t, J = 3.9 Hz, 1H), 7.67 (t, J = 7.5 Hz, 1H), 7.48 (d, J = 4.9 Hz, 1H). ^{13}C NMR (126 MHz, DMSO) δ 182.90 (C, C=O), 135.70 (C), 134.61 (CH), 133.71 (CH), 132.52 (C), 131.61 (CH), 131.42 (CH), 130.55 (C), 130.45 (CH), 130.30 (CH), 129.55 (CH), 128.52 (C), 128.39 (CH), 127.86 (CH), 127.77 (CH), 127.45 (C), 126.95 (C), 125.33 (CH), 124.79 (CH), 122.96 (C), 121.29 (C), 93.12 (C, C≡C), 86.67 (C, C≡C). HRMS (ESI): m/z calculated for $[C_{23}H_{12}OS + H^+]$ 337.0682, found 337.0681.

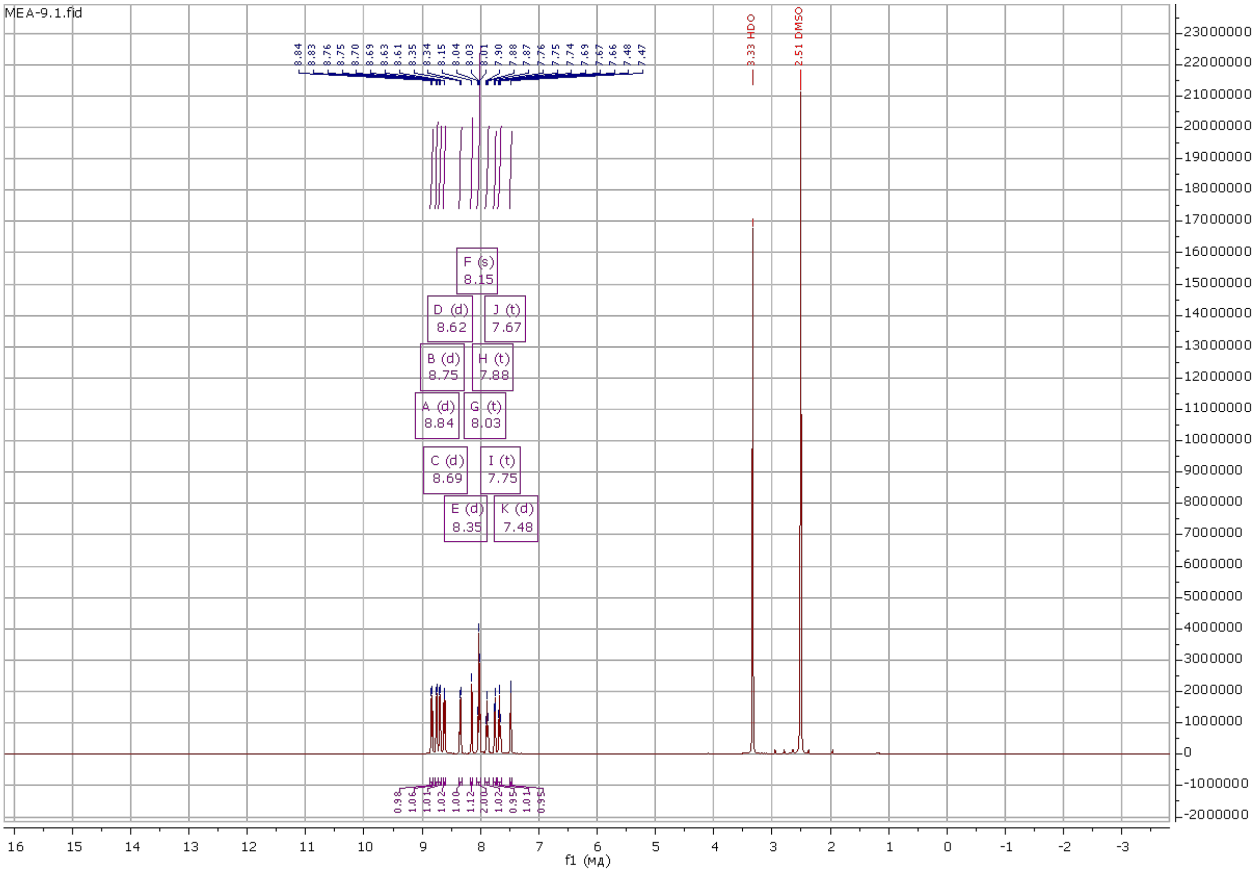

Fig. S4. Dye B ^{1}H NMR spectrum (500 MHz, DMSO-d_6).

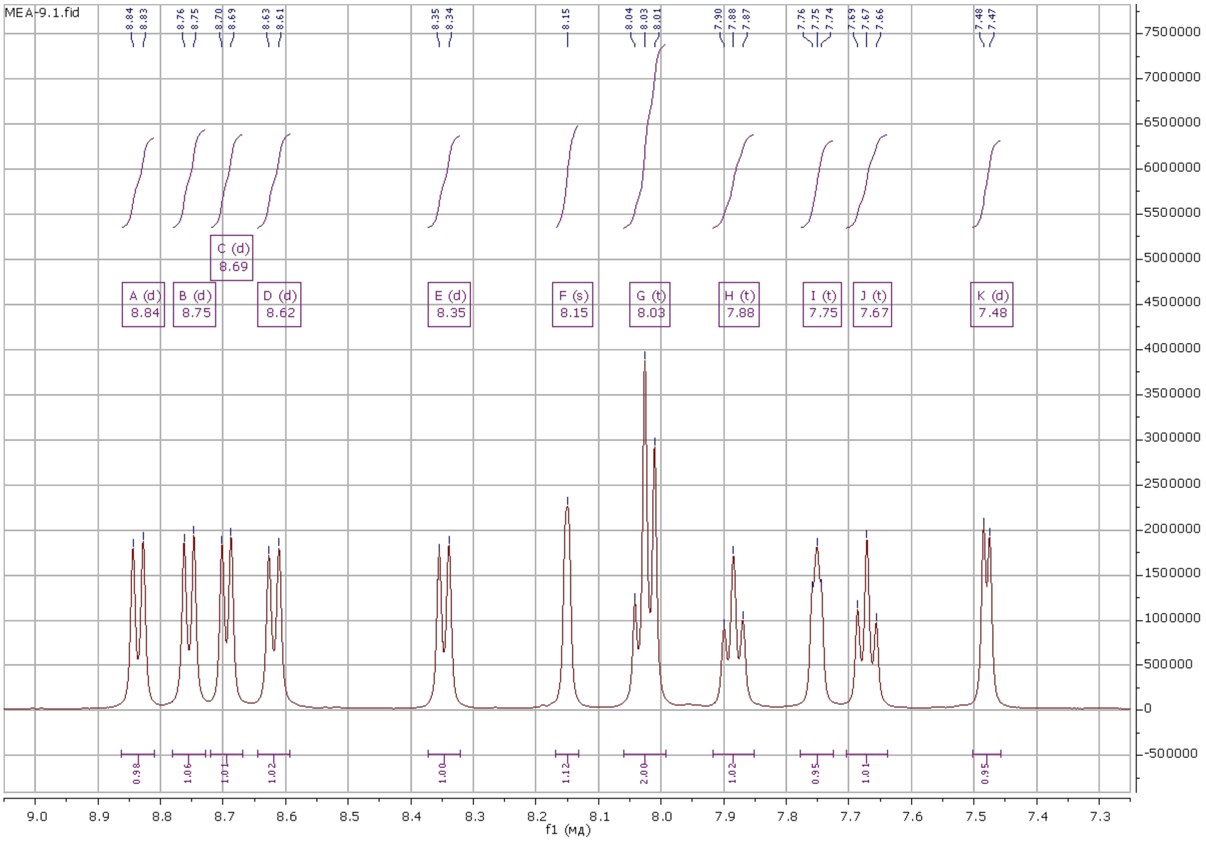

Fig. S5. Dye B ^{1}H NMR spectrum; aromatic region (500 MHz, DMSO-d$_6$).

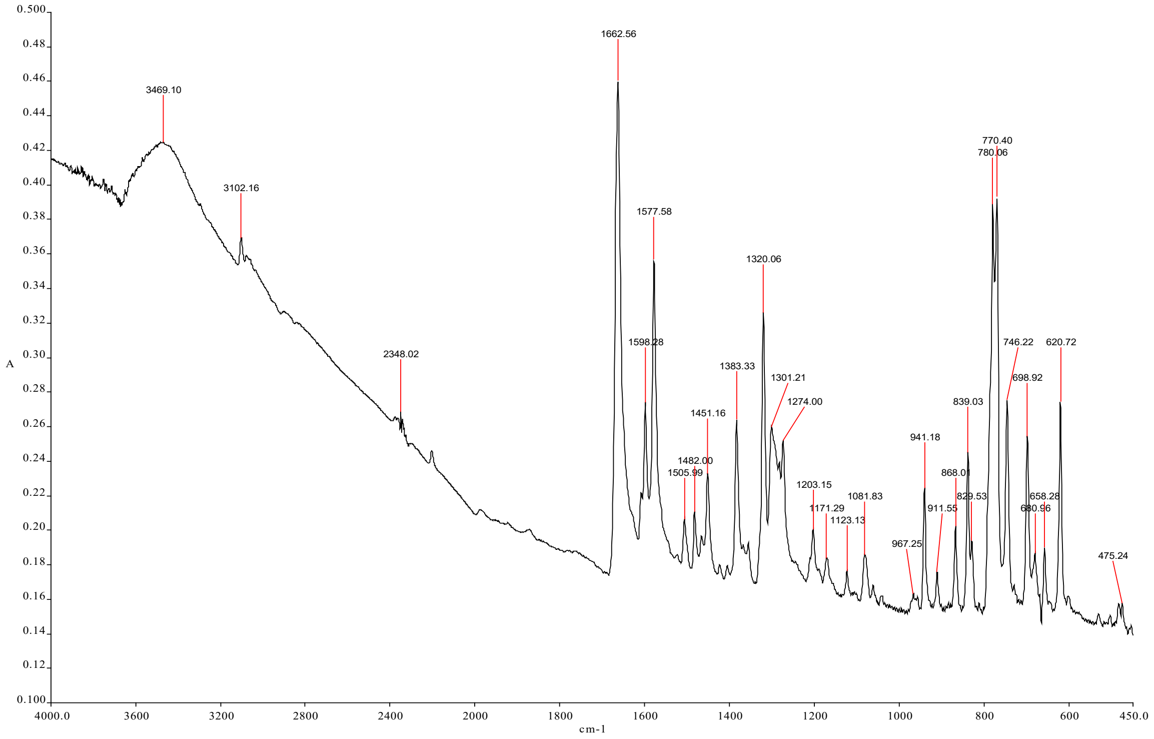

Fig. S6. Dye B IR spectrum.

$[M+H]^+$ calc	$[M+H]^+$ found	Δ
337,0682	337,0681	0,0001

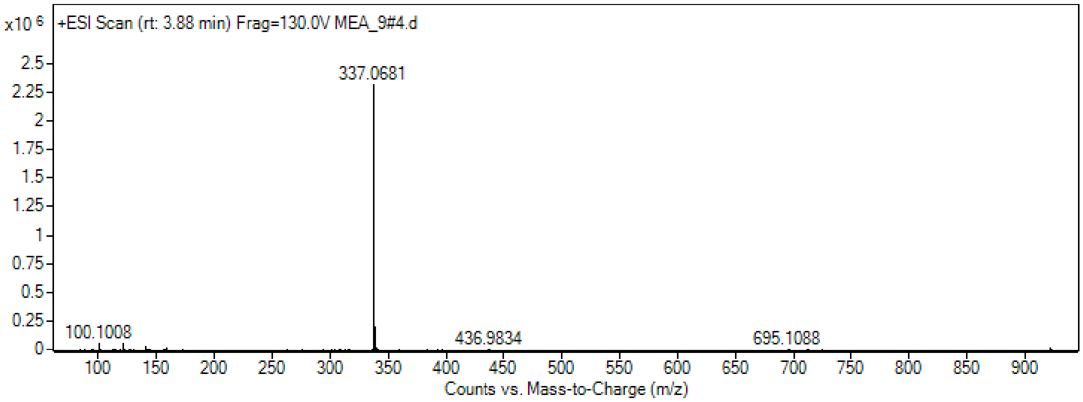

Fig. S7. High-resolution mass spectrum of Dye B.

(Dye C): 3-(3-Methoxy-3-oxoprop-1-yn-1-yl)-7-oxo-7H-benzo[de]anthracene-7-one

SMILES strings: O=C1C2=CC=CC=C2C3=CC=C(C#CC(OC)=O)C4=CC=CC1=C34

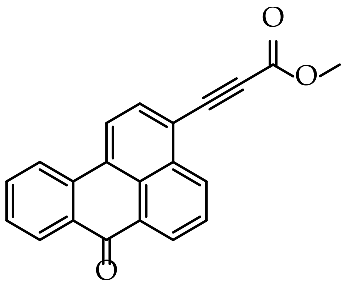

Dark yellow solid. Yield: 61 %. Melting point: 172 °C. R_f = 0.74 (hexane/chloroform/acetone; 4:2:1). IR, λ_{max} (KBr) cm^{-1}: 1652 (C=O), 2207 (C≡C). ^{1}H NMR (500 MHz, DMSO-d_6) δ 8.63 (d, J = 7.3 Hz, 1H), 8.58 – 8.49 (m, 3H), 8.28 (d, J = 7.8 Hz, 1H), 8.07 (d, J = 8.0 Hz, 1H), 7.98 (t, J = 7.8 Hz, 1H), 7.84 (t, J = 7.7 Hz, 1H), 7.64 (t, J = 7.5 Hz, 1H), 2.51 (s, 3H). HRMS (ESI): m/z calculated for $[C_{21}H_{12}O_3 + H^+]$ 313.0859, found 313.0872.

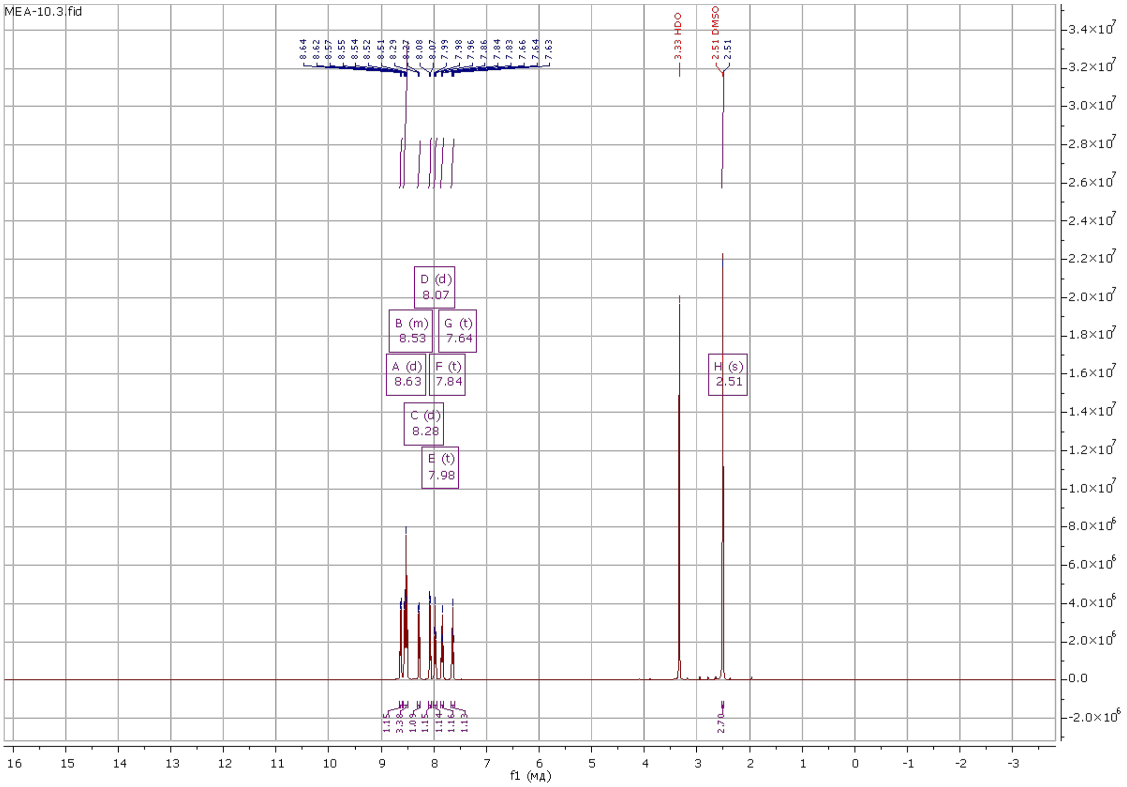

Fig. S8. Dye *C* ^{1}H NMR spectrum (500 MHz, DMSO-d_6).

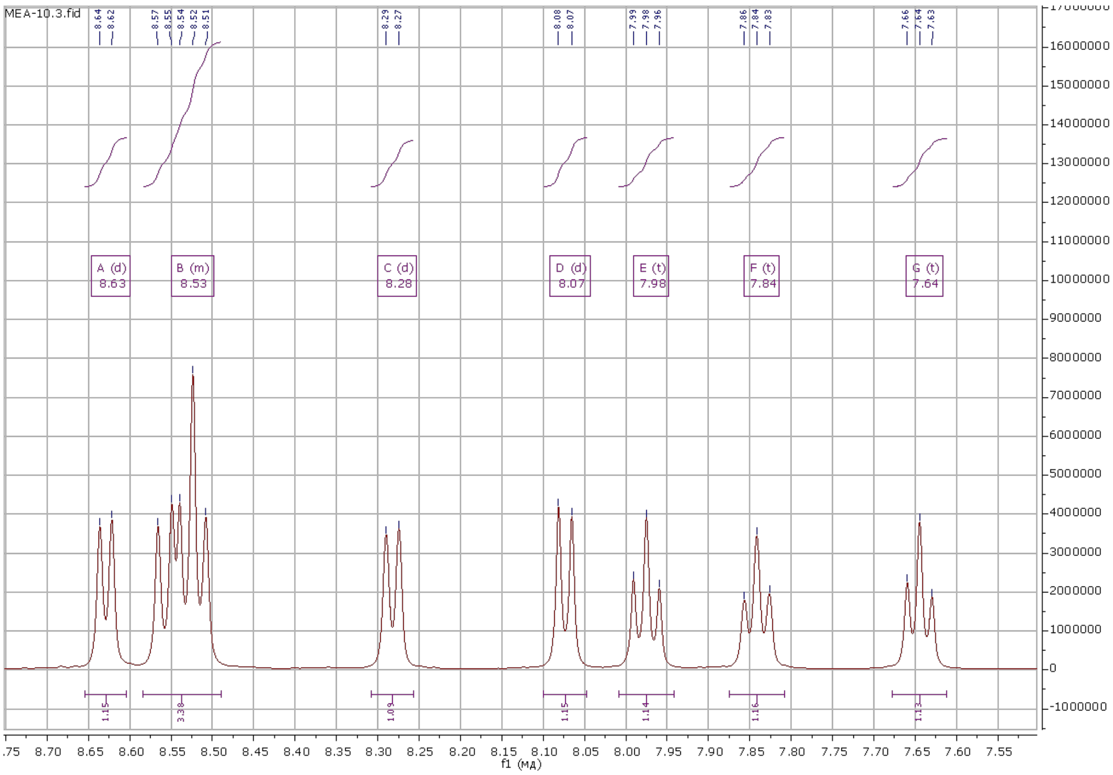

Fig. S9. Dye C ^{1}H NMR spectrum; aromatic region (500 MHz, DMSO-d_6).

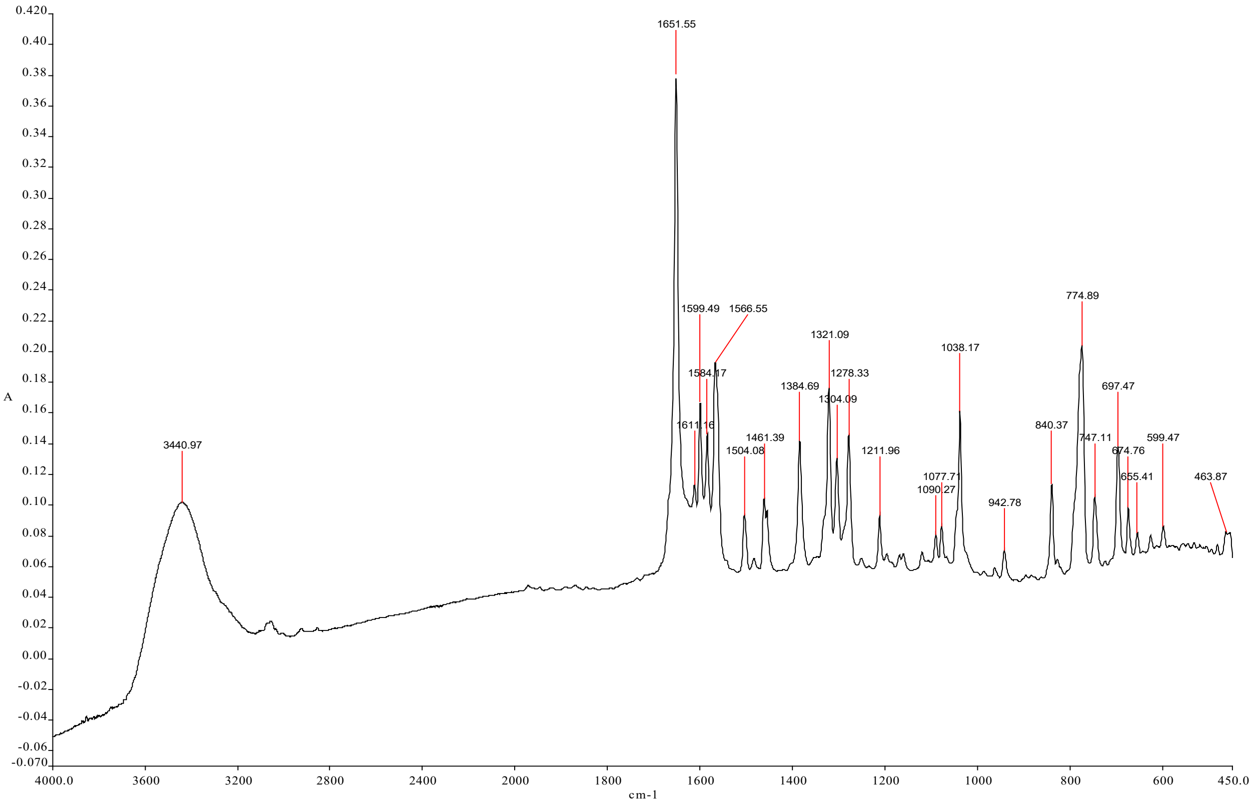

Fig. S10. Dye C IR spectrum.

$[M+H]^+$ calc	$[M+H]^+$ found	Δ
313,0859	313,0872	0,0013

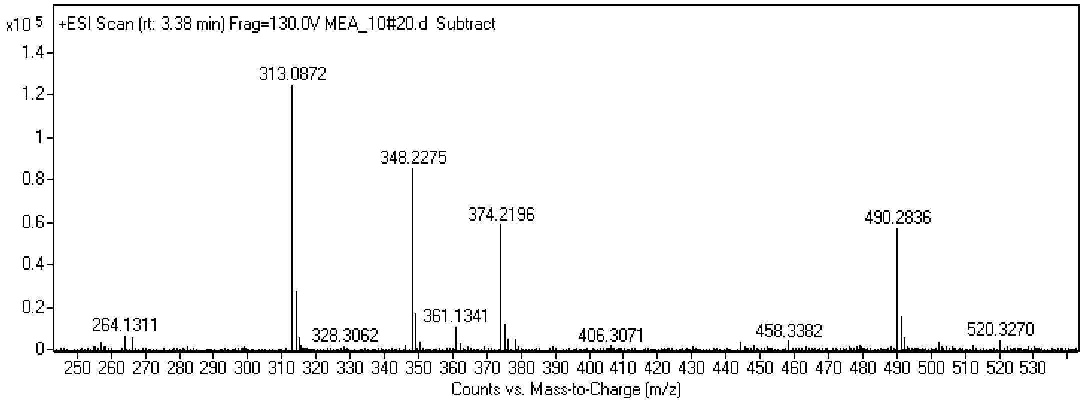

Fig. S11. High-resolution mass spectrum of Dye C.

Table S1: Estimated Static and Dynamic First-order hyperpolarizabilities

Molecule	Static β(0) (10^{-30} esu)	EOPE Dynamic β (-ω; ω,0) (10^{-30} esu)	SHG Dynamic β(-2ω; ω, ω) (10^{-30} esu)
		Wavelength ω = 532 nm	
Dye A	8.236	15.48	294.6
Dye B	13.77	42.316	370.91
Dye C	6.796	12.826	120.792

Table S2. Estimated Static and Dynamic Second-Order Hyperpolarizabilities

	Second order Hyperpolarizability γ (all values should be multiplied by 10^{-36} esu)		
Molecule	Static γ(0)	EOKE γ (-ω; ω,0,0)	SHG γ (-2ω; ω, ω,0)
	Wavelength ω = 0 nm	Wavelength ω = 532 nm	
Dye A	227.21	637.68	-2141.66
Dye B	152.26	417.60	-44629.7
Dye C	78.89	177.53	32.82

Table S3. Calculated isotropic, anisotropic polarizability and refractive indices of Dye A, B, C in DMF solvent.

Molecule	Dye A	Dye B	Dye C
αxx	84.62	54.33	90.77
αyy	51.71	55.08	22.04
αzz	87.18	89.45	51.09
αxy	-42.3	-32.68	13.70
αyz	22.77	23.33	1.08
αzx	-33.30	-23.08	5.62
<α>	74.50	66.29	54.63

10^{-24} esu			
$\Delta\alpha$ 10^{-24} esu	147.22	118.65	69.96
Volume (cm^3/mol)	257.8	246.72	226.57
n	3.01	2.70	2.38
